# Primary and Recurrent Erysipelas—Epidemiological Patterns in a Single-Centre Retrospective Analysis

**DOI:** 10.3390/jcm14155299

**Published:** 2025-07-27

**Authors:** Marta Matych, Agata Ciosek, Karol Miler, Marcin Noweta, Karolina Brzezińska, Małgorzata Sarzała, Joanna Narbutt, Aleksandra Lesiak

**Affiliations:** 1 Department of Dermatology, Pediatric Dermatology and Dermatological Oncology, Medical University of Lodz, 90-419 Lodz, Poland; 2 Laboratory of Autoinflammatory—Genetic and Rare Skin Disorders at Department of Dermatology, Pediatric Dermatology and Dermatological Oncology, Medical University of Lodz, 90-419 Lodz, Poland; 3 Student Scientific Research Club of Experimental, Clinical and Procedural Dermatology, Medical University of Lodz, 90-419 Lodz, Poland; 4 Faculty of Medicine, Pomeranian Medical University, 70-111 Szczecin, Poland

**Keywords:** recurrent erysipelas, bacterial skin infection, risk factors, dyslipidemia, lymphedema, obesity, diabetes

## Abstract

**Background/Objectives:** Erysipelas is an acute bacterial skin infection, particularly affecting the lower limbs, with a tendency to recur. Despite its clinical importance, data on demographic and epidemiological risk factors, as well as factors influencing hospitalization, remain limited. This study aimed to analyze the epidemiological and clinical characteristics of patients hospitalized with primary and recurrent erysipelas, focusing on risk factors contributing to disease onset, recurrence, and prolonged hospitalization. **Methods:** A retrospective single-center analysis was conducted on 239 patients hospitalized for erysipelas at the Department of Dermatology, Pediatric Dermatology, and Oncology at the Medical University of Lodz. Data collected included demographics, lesion location, laboratory markers, comorbidities, and hospitalization outcomes. Statistical analyses were performed to assess associations between risk factors, disease recurrence, and hospitalization duration. **Results:** The majority of erysipelas cases (85.4%) involved the lower limbs, with a higher prevalence in men. Upper extremities were mostly affected in women, especially those who had undergone breast cancer surgery. Recurrent erysipelas accounted for 75.7% of cases. Most patients (89.1%) had at least one comorbidity, with hypertension, diabetes type 2 (DM2), and obesity being the most common. Higher white blood cell (WBC) count, obesity, atrial fibrillation (AF), and the need for enoxaparin administration were independently associated with prolonged hospitalization. Dyslipidemia was significantly associated with erysipelas recurrence (*p* < 0.05). **Conclusions:** Both primary and recurrent erysipelas are associated with specific risk factors. Recurrent erysipelas may be linked to components of metabolic syndrome, particularly obesity and dyslipidemia, which emerged as a significant risk factor in this study. Hospitalization length may be prolonged by inflammation markers (WBC and CRP) and comorbidities such as AF, obesity, or the need for enoxaparin in patients with elevated thrombosis risk. Further multicenter studies with larger cohorts are needed to assess the impact of demographics, biomarkers, metabolic disorders, and treatment strategies on erysipelas recurrence and outcomes. Awareness of these risk factors is essential for effective prevention, management, and recurrence reduction.

## 1. Introduction

Erysipelas is an acute bacterial skin infection, primarily affecting the upper dermis, which spreads through superficial lymphatic vessels [1,2]. The main causative agent of the disease is Group A beta-hemolytic streptococci, with *Streptococcus pyogenes* being the most prevalent pathogen [1,2]. Infections caused by Group C and G streptococci or staphylococci are much less common [1]. In recent years, erysipelas has become increasingly prevalent [3], likely due to an aging population and the rising incidence of comorbid conditions such as diabetes and obesity [4,5,6]. It can affect all age groups but is most frequent among elderly individuals [7]. The main factor contributing to the outbreak of the disease is skin trauma [8], which may include minor injuries such as scratches or interdigital fissures that accompany skin conditions including atopic dermatitis (AD), psoriasis, and fungal infections [9,10,11]. These disruptions to the skin barrier facilitate bacterial penetration [12]. Moreover, postoperative wounds and ulcers also promote the development of erysipelas [13,14]. Additionally, certain chronic conditions—such as diabetes, circulatory diseases (venous insufficiency or lymphoedema), and immune deficiencies—along with alcoholism, are considered risk factors for both primary and recurrent erysipelas [7,15,16]. Outbreaks are usually abrupt in onset and often associated with general symptoms including fever, chills, shivering, and regional lymphadenopathy. While erysipelas most commonly affects the skin of the lower limbs or face, the involvement of the upper limbs or trunk is less common, and areas like the scalp, ear, or genital region are rarely affected [17,18,19]. The affected skin has a very sharp, raised border. Skin lesions are usually bright red, firm, and swollen with blistering or bleeding [8,16]. The lesions are usually accompanied by pain and increased warmth of the affected side [8]. For this reason, the condition is also historically referred to as St. Anthony’s fire due to the intense rash associated with it [2]. This term is also used to describe other distinct diseases, including ergotism and herpes zoster, depending on the region and era. In some historical contexts, even other illnesses such as the plague may have been misclassified under this broad term. While still occasionally used colloquially today, especially for herpes zoster in some countries, the use of “St. Anthony’s fire” should be approached with caution due to its ambiguous and variable historical usage [20]. The diagnosis of erysipelas is primarily clinical, based on its characteristic presentation and the exclusion of other differential diagnoses [8], which include non-infectious conditions such as allergic contact dermatitis, angioedema, erythema nodosum, and infectious erysipeloid, Lyme borreliosis, and herpes zoster [21,22]. Blood tests often reveal an increased white blood cell (WBC) count, usually elevated due to systemic inflammation, often with notable neutrophilia, along with elevated C-reactive protein (CRP) levels—a sensitive marker of acute inflammation—and procalcitonin (PCT), which may help distinguish bacterial from viral or non-infectious causes and tends to rise in severe bacterial infections, especially when erysipelas leads to bacteremia [15,23]. The standard causal treatment involves antibiotic therapy with beta-lactam antibiotics, most notably amoxicillin [24,25]. When bacterial resistance is suspected, it is administered in combination with clavulanic acid [24]. Alternative options include cephalosporins, which should be used with caution in patients with a history of penicillin allergies. In cases of severe penicillin allergy, lincosamides or macrolides are recommended [25]. The choice of antibiotic therapy is crucial as it significantly influences the course and duration of hospitalization and, ultimately, the effectiveness of treatment. However, even when initiated promptly, therapy does not fully prevent relapses, which may occur in up to 30% of patients [26,27]. Other complications of erysipelas include abscess formation, bullous lesions, thrombophlebitis, skin necrosis, hemorrhagic purpura, lower limb changes, and abdominal elephantiasis [2].

Current data on the clinical and epidemiological factors associated with the risk of erysipelas and its recurrence remain scarce. The aim of this study was to investigate the demographic and clinical characteristics of both primary and recurrent erysipelas to raise awareness of relevant risk factors. Understanding the demographic and clinical aspects of erysipelas, particularly its association with comorbidities, is crucial for providing appropriate medical care and reducing the risk of recurrence.

## 2. Materials and Methods

### 2.1. Data Collection Process

We conducted a retrospective single-center analysis of medical records from 239 patients hospitalized due to primary and recurrent erysipelas in the Department of Dermatology, Pediatric Dermatology, and Oncology at the Medical University of Lodz between 2014 and 2023. Patients were identified in the database using the following “ICD-10-CM’s 2024” code: A46 (Erysipelas).

From the database, various parameters associated with the first documented hospitalization were extracted, including (1) year; (2) gender; (3) age; (4) lesion localization; levels of inflammatory markers such as (5) white blood cell (WBC) count, (6) C-reactive protein (CRP), and (7) procalcitonin (PCT) levels; (8) D-dimer levels; (9) body temperature upon admission (°C); number of (10) primary and (11) recurrent cases; and (12) comorbidities. In cases of suspected superficial thrombophlebitis or deep vein thrombosis based on clinical suspicion in elderly, diabetic, obese, or immobile patients (13), Doppler examination was performed, and those patients were administered with a prophylactic dose of enoxaparin (40 mg subcutaneously once daily).

The data were collected by four authors (M.M., K.B., M.S., and A.C.), and tables were prepared using Google Sheets (Google LLC, Mountain View, CA, USA; accessed September 2024) and Microsoft Excel (version 2301, Microsoft Office Professional Plus 2016; Microsoft Corporation, Redmond, WA, USA). Data regarding the recurrence of erysipelas were obtained by checking the follow-up history.

### 2.2. Eligibility Criteria

Our study included only hospitalized patients admitted to the Department of Dermatology, Pediatric Dermatology, and Oncology of the Medical University of Lodz. As not all erysipelas cases require hospitalization, patients with mild or moderate disease managed on an outpatient basis were not included in the study. We included adult patients with a confirmed diagnosis of first-episode or recurrent erysipelas (ICD-10 A46) and complete medical history (subjective and objective examination) with available additional tests (blood work and body temperature). Patients who were transferred to other departments, including internal medicine, due to exacerbation of comorbidities, were included in this study, provided that appropriate treatment for erysipelas was prescribed and administered.

Exclusion criteria for this study included a diagnosis of an alternative differential diagnosis; incomplete medical records (missing documentation of the patient’s examination upon admission, absent results of additional tests such as blood work or body temperature, or no follow-up history); and lack of patient consent for hospital admission, treatment, or early discharge upon request (Table 1).

### 2.3. Statistical Analysis

Statistical analysis was performed by one of the authors (K.M.) using data analysis software system Statistica 13.3 (TIBCO Software Inc., Palo Alto, CA, USA). Quantitative data according to gender were compared using an unpaired *t*-test. The chi-square test was used to assess categorical data group differences. Pearson’s r was used to evaluate the strength and direction of the correlation. The Cox proportional hazards model was applied to investigate the influence of multiple factors on hospitalization length, using time to discharge as the outcome measure in patients with complete follow-up. Statistical significance was defined as *p* < 0.05. Visualizations were generated in R (version 4.4.3) using the ggplot2 package and its extensions, including ggbreak [28] for axis modification.

## 3. Results

### 3.1. General Information

Between 2014 and 2024, a total of 239 cases of primary and recurrent erysipelas were reported, with 52.7% (n = 126) of the cases involving men and 47.3% (n = 113) involving women. The average age of the participants was 64 years, with a mean age of 60 years among men and 68 years among women. The difference in the age of women and men at the time of first hospitalization was statistically significant (*p* < 0.05). Of all patients, 24.3% (n = 58) were diagnosed with primary erysipelas, while 75.7% (n = 181) experienced recurrent episodes, with a mean number of recurrences of 1.5 per patient. The average duration of hospitalization was 9.8 days. No gender-related differences in erysipelas recurrence and time of hospitalization were observed. The characteristics of the patients according to gender are presented in Table 2.

### 3.2. Lesion Localization

Among the 239 patients, 85.4% (n = 204) had lower limb involvement, with the left lower limb being the most commonly affected site (45.2%, n = 108), followed by the right lower limb (38.5%, n = 92). The second most common location was the upper limb (7.1%, n = 17). Other areas (face, chest, and groin) were affected sporadically.

**Figure 1:**
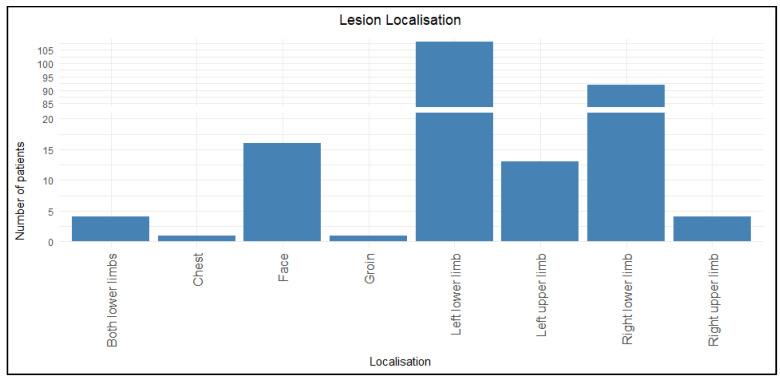
Distribution of lesions among participants.

Figure 1 represents the distribution of lesions among participants. The distribution of the lesions according to gender was also analyzed (Table 3). The difference in location between the genders is statistically significant in both primary and recurrent erysipelas (*p* < 0.05). According to the results, erysipelas of the lower limbs was mainly linked to the male gender, while the upper limb and other locations (face, groin, and chest) were predominant in women.

As previously mentioned, the upper limb, both in the case of primary and recurrent erysipelas, was mostly affected in women (6.7%, n = 16), especially those diagnosed with breast cancer and those who received surgical treatment for it. In 75% (n = 12) of cases, women underwent mastectomy with lymph node dissection or resection of the breast tumor (Table 4). One of the female patients (0.9%, n = 1) developed erysipelas of the chest after mastectomy. In these women, the risk of erysipelas recurrence was also increased compared to the study population and accounted for about 3.3 relapses per patient.

### 3.3. Results of Additional Tests

All patients underwent blood tests and body temperature measurements upon admission. The mean values of the inflammatory markers were as follows: WBC—11.9 × 10^3^/µL; CRP—133.5 mg/L; and PCT—1.98 ng/mL. The average body temperature was 38.1 °C. D-dimer levels were measured in 134 patients (56%), with a mean value of 1710.7 µg/L. Additionally, 76 patients underwent Doppler ultrasound, and venous thrombosis was detected in only one of them (1.3%, n = 1). The results of the statistical analysis of inflammatory markers are shown in Table 5.

**Figure 2:**
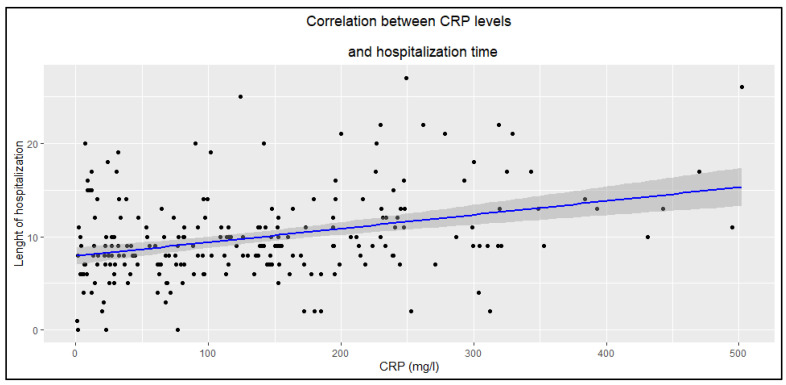
Correlation between CRP levels and hospitalization time (R = 0.33, *p* < 0.0001).

**Figure 3:**
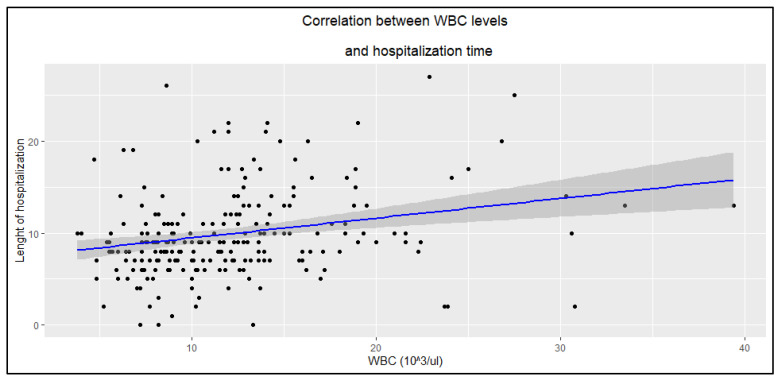
Correlation between WBC levels and hospitalization time (R = 0.25, *p* < 0.0001).

Higher CRP and WBC levels were associated with longer hospitalization duration (Figure 2 and Figure 3), whereas PCT showed no such statistically significant correlation (R = 0.14, *p* = 0.058).

**Figure 4:**
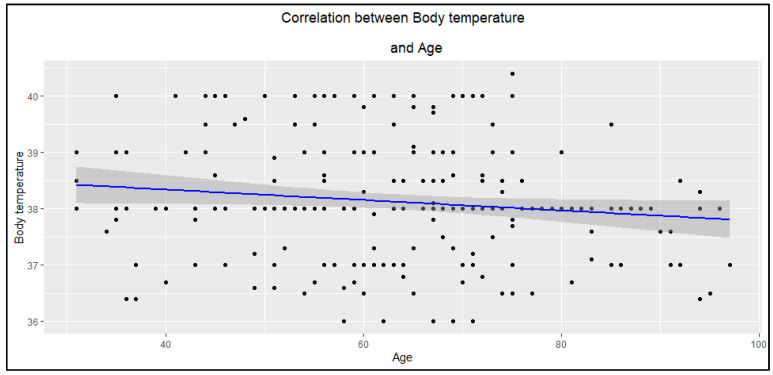
The correlation between body temperature and age of participants (R = −0.13, *p* = 0.046).

Moreover, it was observed that in older patients, the body temperature at the first hospitalization was significantly lower compared to younger patients (Figure 4).

### 3.4. Comorbidities

**Figure 5:**
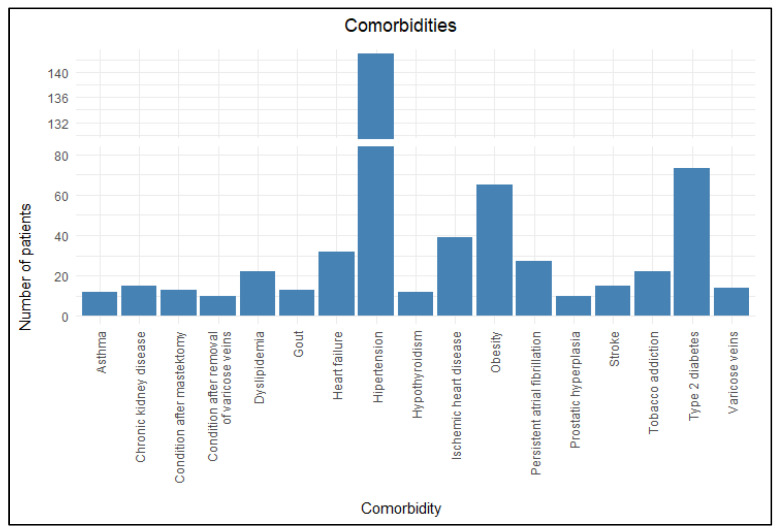
Comorbidities.

Almost all participants included in the study had comorbidities, with as many as 89.1% patients (n = 213) being affected. The average number of comorbid conditions per patient was 3.6. The most common were hypertension (59.8%, n = 143), type 2 diabetes (DM2) (30.5%, n = 73), obesity (27.2%, n = 65), ischemic heart disease (16.3%, n = 39), heart failure (13.4%, n = 32), persistent atrial fibrillation (11.3%, n = 27), and dyslipidemia (9.2%, n = 22). Figure 5 presents the main comorbidities identified in the study.

Among the comorbidities mentioned above, only the prevalence of dyslipidemia differed significantly between the primary and recurrent erysipelas groups according to the chi-squared test (Table 6) and had a statistically significant effect on the number of erysipelas recurrences according to the U-Mann–Whitney test (Table 7), unlike the more common hypertension, DM2, obesity, ischemic heart disease, heart failure, and persistent atrial fibrillation.

### 3.5. Risk of Prolonged Hospitalization

**Figure 6:**
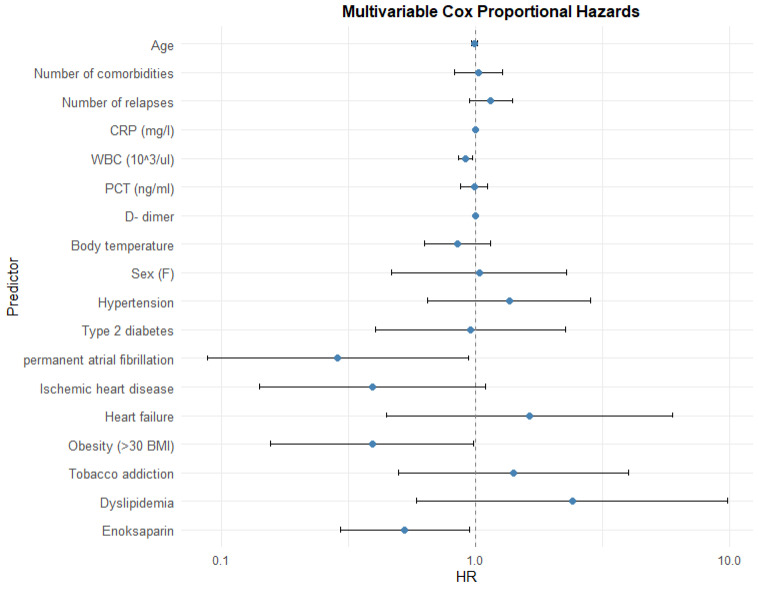
Impact of variables.

The Cox proportional hazard model showed that increased WBC levels, presence of permanent atrial fibrillation (AF), obesity, and the need for enoxaparin administration are independent risk factors of prolonged hospitalization (Table 8; Figure 6). A similar relationship has not been observed for other inflammatory markers. Also, none of the other comorbidities are related to extended hospitalization time.

## 4. Discussion

Erysipelas eludes precise statistics. Its exact incidence is difficult to determine due to the very diverse nature of patient care—some cases are treated on an outpatient basis, while others require hospitalization in different departments, including dermatology, surgery, and infectious diseases [29]. This bacterial skin infection tends to recur [30]. Its recurrence might lead to future complications, including scarring, skin damage, chronic lymphoedema, or even elephantiasis [16]. The available demographic and epidemiological data are limited. This study reports the characteristics of 239 adult patients hospitalized in the Department of Dermatology, Pediatric Dermatology, and Oncology at the Medical University of Lodz due to primary and recurrent erysipelas and evaluates possible risk factors associated with its outbreaks, relapses, and course of hospitalization.

### 4.1. Demographic Characteristics of Patients

Erysipelas affects both genders, and according to our study, it was more prevalent among men (52.7% vs. 47.3%). However, the data available in the literature are inconsistent as to which gender is more susceptible to the disease. The study by Sočan K. and Sočan M., conducted on a group of over 36,000 patients and lasting for 6 years, showed that women were more likely to suffer from erysipelas [31], while Bläckberg A et al. showed that 59% of patients with erysipelas were men [32]. The results indicate that women develop erysipelas at a significantly later age compared to men (68 vs. 60 years old; *p* < 0.05). Despite this, there seems to be no significant gender-related impact on the number of relapses or the length of hospitalization.

### 4.2. Anatomical Distribution of Erysipelas

Lesions were mostly located in the lower limbs (85.4%), especially the left lower limb (45.2%). According to the results, the anatomical distribution of erysipelas was linked to gender. Changes in the lower limb were mostly associated with the male gender, while those on the upper limb and other locations occurred more often in women. The higher frequency of upper limb involvement in women is due to the occurrence of breast cancer and its surgical treatment. Of the 16 women with affected upper limbs, 75% (n = 12) underwent radical mastectomy with lymph node dissection or tumor excision. As a result of these procedures, permanent damage to the lymphatic vessels and disruption of lymph circulation occurs and results in the development of chronic lymphoedema [33], which favors the development and frequent recurrence of erysipelas [34]. In men, erysipelas affects the lower extremities, which is primarily attributed to a higher percentage of nicotine addiction, cardiovascular diseases, obesity-related metabolic consequences, and higher exposure to trauma (e.g., yard work and gardening and extreme sports) [35,36,37]. Men also tend to neglect foot hygiene in chronic diseases like diabetes [38], which might provide the entry point for infection.

### 4.3. Additional Tests

#### 4.3.1. Inflammatory Markers

Higher CRP and WBC levels were associated with longer hospitalization in patients with erysipelas, whereas PCT showed no statistically significant correlation (*p* > 0.05). This may suggest that CRP and WBC better reflect the extent of the inflammatory response, which is markedly elevated in erysipelas [8,15,16], and are correlated with the severity of the episode, while PCT, typically a marker of severe bacterial infections and sepsis, may be less sensitive in localized skin infections and highlights the importance of the possible toxin-mediated etiology of the disease [8]. In this study, out of the 239 patients, blood was collected for bacteriological examination in only 35 cases (14.6%), and bacterial growth was detected in just 1 case—*Streptococcus dysgalactiae*.

#### 4.3.2. D-Dimer Levels

D-dimer levels were measured in 134 patients (56%), with a mean value of 1710.7 µg/L. Although elevated D-dimer levels are commonly associated with a higher risk of thromboembolic events, our study did not demonstrate the occurrence of thrombosis in patients (thrombosis was detected only in one individual during Doppler examination). Additionally, no significant correlation was found between the D-dimer levels and the duration of hospitalization or the rate of recurrence in patients with erysipelas. Nevertheless, its elevation remains clinically relevant. Given that erysipelas is an acute inflammatory condition, transient increases in D-dimer levels are expected [39]. Persistent or markedly elevated levels should prompt clinicians to consider additional diagnostic evaluation for thrombotic complications, especially in elderly patients or those with pre-existing risk factors. Therefore, while D-dimer levels were not predictive of clinical outcomes in our cohort, it remains an important biomarker that warrants attention in the broader context of patient safety.

#### 4.3.3. Body Temperature

The majority of patients had fever upon admission to the hospital, and the average temperature was 38.1 °C. According to the study results, older age was not associated with a significant increase in body temperature or the occurrence of fever as observed in younger patients. This is because in older patients, the inflammatory reaction and infections are often less pronounced and may proceed in an atypical manner [40,41]. However, the clinical condition of these patients should not be underestimated as it does not necessarily correlate with the severity of infection in a given episode, and serious cases may even lead to severe systemic complications such as sepsis.

### 4.4. Risk Factors of Outbreaks, Recurrences, and Prolonged Hospitalization

#### 4.4.1. Risk Factors of Primary Erysipelas

Although our study did not reveal any statistically significant characteristics influencing the occurrence of outbreaks and primary erysipelas, we may find data concerning such factors in the literature. The main cause of primary erysipelas is skin trauma, which may be caused by dermatological conditions, such as athlete’s foot and nail fungus, or due to comorbidities such as chronic venous insufficiency, ulcerations of the lower limbs, being overweight, local edema, or lymphedema [42,43].

#### 4.4.2. Comorbidities as a Risk Factor of Recurrent Erysipelas

According to the literature, recurrences of erysipelas are linked to various risk factors. The main ones include obesity, DM2, lymphedema, and venous insufficiency [7,16,26,44]. Erysipelas commonly affects patients with comorbidities that facilitate the development of the disease. In our study, 89.1% of patients were burdened with at least one underlying condition. However, the number of comorbidities did not influence the length of hospitalization nor the number of recurrences. An additional area of interest is the link between recurrence and components of metabolic syndrome, which, apart from obesity and diabetes, also includes hypertension [16,29]. All of the above were the most common diseases accompanying patients in the study group. Although this study did not show any significant correlation between the primary or recurrent erysipelas and the components of the metabolic syndrome, each one of the diseases affects factors related to the pathomechanism of the disease, such as impaired blood and lymph circulation, micro- and macroangiopathies, neuropathies (especially in patients suffering from DM2), and reduced immunity. Hypertension contributes to impaired blood circulation. This predisposes patients to chronic venous stasis, tissue edema, and impaired microcirculation [45]. These conditions might weaken skin integrity, disrupt lymphatic drainage, and impair local immune defense, facilitating bacterial invasion and infection [46]. In a study by Kozłowska et al., one of the main diseases accompanying patients with recurrent erysipelas, along with obesity, was hypertension [29]. DM2 is one of the main factors that impairs the healing process [47]. Hyperglycemia reduces the neutrophil and monocyte immunological functions (such as chemotaxis, adherence, and phagocytosis) [48,49], and because of that, patients with diabetes are at greater risk of infection, including group A and B streptococci and Staphylococcus aureus [50]. Additionally, diabetes causes peripheral neuropathy, which contributes to sensory disorders, especially in the lower limbs, as well as injuries [51]. Consequently, DM2 is a relevant risk factor for primary and recurrent erysipelas [15,16]. Impaired lymphatic circulation is the primary factor underlying the development of erysipelas. Sudduth C. and Greene A. from Harvard Medical School highlighted that obesity itself is one of the most important factors significantly increasing the risk of lymphedema, and they proposed naming this condition obesity-induced lymphedema (OIL). Obesity predisposes individuals to lymphatic circulation disorders because of increased inflammation, which destroys vessels, weakens muscle contractions, and places excessive pressure on vessels [52]. Scientific papers highlight obesity as an important factor in the development of erysipelas [26,29,53], especially in the lower limbs [54].

Our study demonstrated a significant correlation (*p* < 0.05) between dyslipidemia and the recurrence of erysipelas. Dyslipidemia contributes to lymphatic dysfunction by altering the composition of lymph, increasing its viscosity, and impairing its drainage [55]. This leads to chronic lymphatic stasis and persistent tissue edema, which are well-recognized risk factors for skin barrier disruption and bacterial entry. Additionally, lipid metabolism disorders are linked to low-grade systemic inflammation and endothelial dysfunction, which further compromise microcirculation and local immune responses [56,57]. These combined mechanisms create a favorable environment for recurrent skin infections, including erysipelas. According to the literature, dyslipidemia is still a poorly understood risk factor for recurrent erysipelas and cellulitis compared to obesity, diabetes, lymphedema, and venous disease. In a case series by Norimatsu et al., hyperlipidemia was identified as a relevant risk factor for recurrent cellulitis [58]. In a retrospective study conducted among the Japanese population between 2005 and 2018, patients with multiple hospitalizations for cellulitis had significantly higher rates of hyperlipidemia, along with hypoalbuminemia, hypertension, and lymphedema, compared to those hospitalized only once [59]. Additionally, in patients with cancer-associated lymphedema who underwent liposuction, factors such as prior erysipelas, hyperlipidemia, severe distal limb edema, and older age were significant risk factors for post-surgical recurrence [60].

Despite the fact that many studies indicate a link between the recurrence of erysipelas and the components of metabolic syndrome, not all authors agree on this thesis [61]; therefore, there is a need to further investigate this relationship.

#### 4.4.3. Comorbidities Influencing Hospitalization Time

According to the study results, the main factors correlated with prolonged hospitalization are AF, enoxaparin administration, and obesity. AF often coexists with other cardiovascular conditions and increases the risk of complications such as heart failure or thromboembolism [62,63]. Those conditions can result in a severe course of erysipelas episode, prolonged recovery time, and prolonged hospital stay. The need for enoxaparin, an anticoagulant used to prevent or treat blood clots, indicates a higher risk of thrombotic complications or existing coagulation disorders, requiring careful monitoring and longer hospitalization to manage potential bleeding risks and ensure safe treatment. Obesity affects drug pharmacokinetics and tissue penetration, complicates wound healing, and increases the risk of infections and other comorbidities, all of which can extend the duration of hospital stay [64,65]. Additionally, obesity may impair mobility and rehabilitation [66], further delaying discharge.

### 4.5. Study Limitations

While this study provides valuable insights into the demographic and epidemiological characteristics of patients hospitalized for primary and recurrent erysipelas, certain limitations should be considered when interpreting the findings. This was a single-center, retrospective study conducted on a relatively small and homogeneous group of participants. Furthermore, this study included only hospitalized patients with erysipelas. Since many mild to moderate cases are treated in outpatient settings, our cohort may overrepresent more severe cases. This selection criteria could contribute to selection bias and limit the generalizability of the findings to the broader erysipelas population. Furthermore, the types of outpatient and inpatient treatments administered, which may affect the course and relapses of erysipelas, were not analyzed. It is also important to note that the study population consisted mostly of patients admitted to the dermatology department, while those treated in infectious disease or surgical departments were not included.

### 4.6. Future Perspectives

To improve the external validity and clinical relevance of existing findings, future research should prioritize large-scale, multicenter studies conducted across diverse geographic regions and healthcare systems worldwide. These studies should include both inpatient and outpatient populations to ensure a comprehensive understanding of erysipelas across varying severities and care settings. Particular attention should be given to the influence of demographic variables (including age, sex, socioeconomic status, and ethnicity), systemic biomarkers of inflammation and infection, underlying metabolic disorders (such as obesity, diabetes, and dyslipidemia), and the impact of differing therapeutic interventions and prophylactic strategies on disease recurrence and clinical outcomes. A more nuanced understanding of these factors may contribute to the development of targeted prevention strategies, optimized treatment protocols, and individualized patient management plans. Ultimately, such efforts could significantly reduce recurrence rates, shorten hospitalization durations, and enhance both clinical outcomes and overall quality of care for patients affected by erysipelas.

## 5. Conclusions

Erysipelas remains a clinically significant skin infection with a propensity for recurrence, which contributes to increased morbidity and healthcare utilization. This study highlights the potential role of metabolic syndrome components including hypertension, dyslipidemia, obesity, and type 2 diabetes mellitus in influencing both the recurrence and duration of hospitalization in affected patients. These findings underscore the importance of comprehensive risk stratification and the integration of metabolic disorder management into erysipelas’ care pathways. To substantiate these observations and inform evidence-based preventive and therapeutic strategies, well-designed multicenter studies with larger and more diverse patient populations are essential.

## Figures and Tables

**Table 1:** Eligibility criteria.

Inclusion Criteria	Exclusion Criteria
ICD-10 code A46	Recognition other than erysipelas
Hospitalized adult patients	Patients treated on an outpatient basis
Complete patient history and follow-up history (including examination, blood tests, and body temperature) available at the Department of Dermatology, Pediatric Dermatology, and Oncology	Incomplete medical records Lack of patient’s consent for hospitalization, treatment, or early discharge upon request

**Table 2:** Patient characteristics according to gender.

Variable	All n = 239	Women n = 113 (47.3%)	Men n = 126 (52.7%)	*p* *
Average age	64	68	60	<0.0001
Relapse rate n (%)	181 (75.7%)	82 (72.6%)	99 (78.6%)	0.280
Average hospitalization time	9.82	9.66	9.98	0.427

* *p*-value from unpaired *t*-test.

**Table 3:** Distribution of lesions according to gender.

Localization	Men n (%)	Women n (%)	*p*
	Primary erysipelas		
Upper limb	0 (0%)	2 (3.4%)	0.037
Lower limb	26 (44.8%)	22 (37.9%)	
Other *	1 (1.7%)	7 (12.1%)	
	Recurrent erysipelas		
Upper limb	1 (0.6%)	14 (7.7%)	0.0004
Lower limb	93 (51.4%)	63 (34.8%)	
Other *	5 (2.8%)	5 (2.8%)	
	All		
Upper limb	1 (0.4%)	16 (6.7%)	<0.0001
Lower limb	119 (49.8%)	85 (35.6%)	
Other *	6 (2.5%)	12 (5.0%)	

* Other location: face, groin, or chest; *p*-values obtained from chi-squared test.

**Table 4:** Comparison of occurrence of upper limb erysipelas among women.

	Surgical Treatment of Breast Cancer	Without Surgical Treatment	*p* *
Upper limb	12	4	<0.0001
Lower limb	4	81	

* *p*-values obtained from chi-squared test.

**Table 5:** Characterization of inflammatory markers.

	Mean (±SD)	Minimum	Maximum
CRP	133.5 (±107.3)	1.2	502.2
WBC	12.0 (±5.5)	3.8	39.40
PCT	2.0 (±7.5)	0.02	86.7
D-dimer	1710.7 (±1676.2)	168	9977

**Table 6:** Prevalence of primary and recurrent erysipelas according to comorbidities.

Comorbidity		Primary	Recurrent	*p* *
Hypertension	With	37	106	0.480
	Without	21	75	
Type 2 diabetes	With	20	53	0.454
	Without	38	128	
Obesity	With	15	50	0.793
	Without	43	131	
Ischemic heart disease	With	7	32	0.322
	Without	51	149	
Heart failure	With	7	25	0.737
	Without	51	156	
Persistent atrial fibrillation	With	9	18	0.252
	Without	49	163	
Dyslipidemia	With	1	22	0.024
	Without	57	160	

* *p*-values obtained from chi-squared test.

**Table 7:** Correlation between comorbidity and relapse rate.

Comorbidity	Number of Patients		Difference in Relapse Rate	
	With	Without	U	*p* *
Hypertension	143	96	6837	0.960
Type 2 diabetes	73	166	5909	0.761
Obesity	65	174	5387	0.574
Ischemic heart disease	39	200	3542	0.365
Heart failure	32	207	3134	0.625
Persistent atrial fibrillation	27	212	2497	0.249
Dyslipidemia	22	217	1577	0.009

* *p*-values obtained from U-Mann–Whitney test.

**Table 8:** Multivariable Cox proportional hazards.

Predictor	HR	95% CI		*p*
		Lower	Upper	
Age	0.99	0.97	1.02	0.515
Number of comorbidities	1.03	0.83	1.27	0.805
Number of relapses	1.15	0.95	1.40	0.160
CRP (mg/L)	1.000	0.996	1.003	0.897
WBC (10^3^/uL)	0.91	0.86	0.97	0.006
PCT (ng/mL)	0.99	0.87	1.12	0.856
D-dimer	1.000	0.999	1.001	0.458
Body temperature	0.85	0.63	1.14	0.283
Sex (F)	1.03	0.47	2.28	0.933
Hypertension	1.36	0.65	2.85	0.417
Type 2 diabetes	0.96	0.40	2.27	0.919
Permanent atrial fibrillation	0.29	0.09	0.94	0.039
Ischemic heart disease	0.39	0.14	1.10	0.075
Heart failure	1.63	0.45	5.96	0.459
Obesity (>30 BMI)	0.39	0.16	0.98	0.046
Tobacco addiction	1.41	0.50	4.00	0.514
Dyslipidemia	2.40	0.59	9.85	0.222
Enoxaparin	0.53	0.29	0.95	0.033

## Data Availability

The data presented in this study are available upon request from the corresponding author due to restrictions regarding patients’ privacy.

## Abbreviations

The following abbreviations are used in this manuscript:

WBC	White blood cell
CRP	C-reactive protein
PCT	Procalcitonin
DM2	Diabetes mellitus type 2
AF	Atrial fibrillation
OIL	Obesity-induced lymphedema
HR	Hazard ratio
CI	Confidence interval
R	Pearson’s correlation coefficient
SD	Standard deviation
U	Mann–Whitney U test
